# Left ventricular remodeling and the athlete’s heart, irrespective of quality load training

**DOI:** 10.1186/s12947-016-0088-x

**Published:** 2016-11-17

**Authors:** Giorgio Galanti, Laura Stefani, Gabriele Mascherini, Valentina Di Tante, Loira Toncelli

**Affiliations:** Department of Experimental and Clinical Medicine Sport Medicine and Exercise Unit, University of Florence, AOU Careggi–Italy, Via delle Oblate 5, Florence, Italy

**Keywords:** Athlete’s hearth, Sports activity, Echocardiography

## Abstract

**Background:**

Regular physical exercise determines a progressive increase of the cardiac mass known as adaptive hypertrophy. Up to now, two morphological echocardiographic heart patterns of athletes have been described by Morganroth in 1975: predominant augmentation of wall thickness, and major cavity size in chamber dimensions in the case of prevalent static or dynamic components. The aim of the study was to follow up the impact of physical training on heart morphology and function in a group of elite soccer and rugby players for at least five years.

**Method:**

From January 1993 to December 2015 a group of 250 elite soccer players and 114 rugby players were examined: 78 soccer players and 60 rugby players were followed up for 5 years. They were matched with a control group.

**Results:**

LV dimensions and LVMi were significantly higher in the athletes than in the inactive subjects (LVMi : 123.45; LVMi: 81.5 vs 94.36 g/m2 respectively). After the five-year follow up the athletes showed no significant modifications in cardiac dimensions: (LVDd from 52.00 ± mm to 52.90 ± mm; LVSd increased from 31.58 ± mm to 32.33 ± mm; Left Ventricular CMI from 120.77 to 121.45 g/m2;p = NS in soccer; from 50.43 ± mm to 52.22 ± mm; Left Ventricular Systolic diameter increased from 32.51 ± mm to 32.8 ± mm; Left Ventricular Mass index from 81,5 to 87,4 g/m2;p = NS and no significant enhancement of the aortic root diameter was observed (Aortic root: from 27.39 mm to 31.64 mm in soccer players; from 30,68 mm to 30.95 mm).

**Conclusions:**

No significant differences were found among the athletes practicing sports with different workload components, and resistance training. In trained athletes the dimensions of the LV chamber and LVMi are generally within the upper limits of the normal range. After a five-year follow-up, the dimensions of the chambers of the heart remain within the normal range, despite being within the the upper limits. Regular physical exercise induces mild LV hypertrophy which therefore can be considered an adaptive consequence to stress-exercise.

## Background

Regular and intensive physical exercise induces several morphological and functional heart modifications, characterizing the so-called “athlete’s heart”. This physiological “adaptive” myocardial hypertrophy is related to the intensity and kind of sport practiced and its principal characteristic is represented by a potential reversibility, when the sports activity is reduced or stopped [1, 2].

The normal upper limits of the athlete’s heart are largely derived from echocardiographic evaluation and the reference values in adult athletes are currently reported in literature [3]. Up to date, two morphological echocardiographic heart patterns of athletes have been described by Morganroth: predominant augmentation of wall thickness, and major cavity size in chamber dimensions in the case of prevalent “static or dynamic” components, respectively [1]. The existence of these two main different types of athletes’ heart, both deriving from two different types of training, has been more recently revisited by Spence [3]. The LV remodelling pattern, observed by MRI, shows substantially similar aspects of the cavity size and wall thickness, both in the case of static as well as in dynamic training [3].

The impact of these sports at different load components on the cardiac morphology and function, during long-term sports training, has not yet been well documented.

The aim of the present investigation is to verify the effects of sports training on the morphology of the heart in elite soccer and rugby players over a period of five years. Soccer and rugby are two of the most commonly practiced sports, respectively characterized by a “high-dynamic, low static” and “medium-dynamic, medium static” component.

## Methods

### Subjects studied

From January 1993 to December 2011 we studied an all-male Caucasian group of 252 elite soccer players and 114 elite rugby players (mean age 24.7 ± 5.6). The athletes were evaluated at the beginning of the seasonal period and 78 of the soccer players and 60 rugby players underwent echocardiographic follow-up for 5 years. Both groups of athletes maintained the same lifestyle. The athletes were matched with 200 inactive subjects (IS). Following the actual sports classification [4] both the athlete’s groups, soccers and rugbyst can be considered in the context of mixed exercise despite with a slight different of static and dynamic component of the training load. All the athletes were regularly investigated in order to control the complete abstinence of illicit substances assumption. The nutrition habits were constantly supervised and adherent to the correct mediterranean diet.

### Training modality

Physical demands in soccers and rugbysts can be globally considered mixed, however while soccer request an high dynamic but low static component, rugby on the contrary shows a prevalent strength and power aspects and therefore static component is prevalent. The regular season runs for 11 months a year, during which a stage of 3 weeks with 2 training sessions per day is generally expected in July. A competitive week for soccer players consists of six training sessions and a match. Five workouts, predominantly aerobic, with a sub-maximal intensity [5] (on average 70-80% maximum) are carried out mainly on the field. At mid-week a particular workout is expected: part of this program was held in the field and another in the gym where resistance training was performed, consisting in a total-body program, in particular for the lower limbs at peak muscle power (around 50% maximum), without the use of additional weights to that of the body.

The global seasonal training program for rugby, was substantially similar if compared to the soccer players. Despite this, the specific component of the physical effort for rugby players was in term of static component, higher with respect of the dynamic module. This last component was maintained at moderate level. The seasonal program for rugby training session is normally structured to encourage greater muscle strength than aerobic and to this effect many training sessions provide an exercise programme with weight lifting in the gym and sub-maximal aerobic effort in the field.

All athletes were evaluated yearly at the beginning of the season at the Sports Medicine Department of the University of Florence – Italy. A complete history was taken to exclude any eventual familiarity for chronic metabolic diseases and any assumption of illegal substances. This aspect was verified by the specific and official doping analysis.

#### Anthropometrics and body composition parameters

For the anthropometric parameters, body mass index (BMI kg/m2) measurement was calculated by the formula weight in Kg/height in m^2^. The body surface area (BSA in m^2^) was obtained using the formula of DuBois and DuBois for BSA (0.007184 x (Height (cm) 0.725x Weight (kg) 0.425) [6].

Skin fold measurement is a common method for determining body fat composition by the Durnin & Wormsley formula, therefore the sites investigated were triceps, biceps, sub scapular and supra iliac [7]. Hydration status was analyzed by bio impedance methods, Resistance and Reactance were recorded at rest condition and without physical activity in the previous 12 h [8].

#### Echocardiographic study

The echocardiographic study was conducted by two experienced cardiologists using an echocardiograph MyLabSeven-Esaote,equipped with a probe of 2.5 MHz. Despite the normally work together, an interobserved variability has been calculated by Blandt-Altman plot. As usual a little sample of athletes and subjects was randomly revaluated by the same investigator and the same subset was evaluated by the other observer blinded to the results obtained by the main investigator.

According to the ASE (2011) guidelines [9] all the systo-diastolic LV parameters were calculated at rest conditions. The interventricular septum (IVS), posterior wall thickness (PW), diameter of the left ventricle in end-diastolic (LVEDd), diameter and end-systolic (LVESd), diameter, the right ventricle diameter (RV), the size of the aortic root (AOR) and left atrium antero-posterior dimensions were calculated from the parasternal long-axis view. The assessment of left ventricular mass index (LVMI g/m2) was obtained according to the formula of Devereux; the cardiac mass was indicized also with lean mass [10]. Considering the regularity of the geometry of the left ventricular chamber of the athletes, the ejection fraction (EF) (%) was calculated according to the formula (LVEDd - LVESd / LVEDd), for which the volumes are assembled to the diameter. No additional data regarding the volume of the chamber have been therefore considered important or mandatory in this context and particularly in presence of the athlete’s heart where a regular morphology is normally maintained.

The LV Relative Wall Thickness (RWT) was determined as the ratio of wall thickness and end-diastolic diameter according to the formula: [2x (PWTd + IVSd)/LVDd]. A RWT value of 0.40 was considered the cut-off point [11].

The analysis of the diastolic parameters was performed in the presence of a stable RR interval and in three different but sequential measurements from the four-chamber view. It consisted in the measurements, by Doppler analysis, of the standard diastolic parameters as transmitral flow of E wave and A wave peak velocities, isovolumetric relaxation (IVRT), deceleration time (DTc) and E/A ratio. In addition, in order to complete the diastolic analysis, the TDI parameters, calculated at the basal segment of interventricular septum and the lateral wall of the left ventricle, were also measured at least at the onset of the team enrolment. The analysis of the distolic function was completed by the measure of the Left atria (LA) area and LA volume in order to exclude any potential diastolic dysfunction. After confirmation that these parameters were normal, these last additional measurements were not routinarly repeated during the periodical follow up. All the data, reported as mean as SD, were indexed for the body surface area. From the short axis view for the great vessel, the pulmonary pressure was also measured. An eventual comparison of this value with the tricuspid regurgitation velocity parameter, as expression of the right ventricle pressure, was also possible.

From the five-chamber view, an eventual presence of valve insufficiency was determined by continuous wave and colour Doppler analysis. According to the ACC/AHA Guidelines, valve insufficiency was, just in case, described as the extent of the regurgitant flow on a 0 to 4+ scale, using the colour-flow mapping method.

#### Statistical analysis

A two-way mixed ANOVA model was used to determine the effect of exercise training modalities on measures of cardiac morphology, aerobic fitness, body composition and strength. Statistical significance was assumed at *P <* 0.05. Student’s *t* test was conducted *post hoc* and reported, as indicated by significant ANOVA results. Data are expressed as means ± SD. Comparison of change scores was undertaken using *t* tests.

The interobserver of the data variability was calculated by Bland Altman test. A value of 95 ± 2 *s* of the mean difference has been considered acceptable.

#### Ethical approval

All study procedures were approved by the Human Research and Ethics Committee of the University of Florence. Written, informed consent was obtained from all subjects in writing, and the studies conformed to the *Declaration of Helsinki*.

## Results

### General data

All data are expressed as mean standard deviation (SD). The subjects analyzed were similar in age. The body mass index (BMI) and body surface area (BSA) were substantially similar within the two groups of athletes, however they were significantly higher in athletes if compared to IS (Tables 1, 3, and 5).

**Table 1:** Comparison between the values in the soccer players with a group of IS

	Soccer (A)	IS (B)	*P* Value
	*n* = 250	*n* = 200	
Age (years)	24,7 ± 5,6	26,9 ± 3,7	NS
Height(cm)	180,0 ± 6,3	174,9 ± 5,8	<0,05*
Weight (kg)	75,5 ± 7,7	80,3 ± 12,2	NS
BMI (kg/m^2^)	23,3 ± 1,8	26,3 ± 3,1	<0,05*
BSA (m^2^)	1,95 ± 0,1	1,9 ± 0,2	<0,05*
SBP (mmHg)	119,1 ± 7,4	127,2 ± 11,5	<0,05*
DBP (mmHg)	75,4 ± 6,1	78,8 ± 1,4	<0,05*
HR (b/min)	61,1 ± 9,2	78,8 ± 1,9	<0,05*
IVS (mm)	10,1 ± 1,0	9,3 ± 0,7	<0,05*
PW (mm)	9,8 ± 1,5	9,2 ± 1,1	<0,05*
LVEDd (mm)	53,3 ± 3,7	49,4 ± 5,6	<0,05*
LVESd (mm)	33 ± 3,1	30,9 ± 3,6	<0,05*
LVM (g)	187,1 ± 40,6	153,4 ± 32,1	<0,05*
LVCMi (g/m^2^)	97,5 ± 25,6	78,8 ± 16,6	<0,05*
AoR	30,8 ± 1,9	29,5 ± 2,2	NS
EF%	67,2 ± 9,5	62,5 ± 8,4	NS
Left Atrium	34,8 ± 6,1	31,5 ± 7,6	NS
RV (mm)	23,1 ± 3,5	22,3 ± 2,6	NS
PP (mmhg)	18,2 ± 2,5	19,2 ± 2,2	NS
LA Area (cm^2^)	19,3 ± 3,4	17,3 ± 1,5	NS
LA volume (ml)	59,7 ± 4,6	44,3 ± 0,7	NS
MAPSE (mm)	21,8 ± 1,7	18,2 ± 2,6	NS
TAPSE (mm)	28,8 ± 5,8	25,1 ± 2,5	NS
E^1^ (cm/s)	12,2 ± 2,1	14,0 ± 2,4	NS
A^1^ (cm/s)	8,3 ± 2,7	7,3 ± 1,0	NS
S (m/s)	8,3 ± 1,7	8,2 ± 1,0	NS
E peck (cm/s)	63,1 ± 5,1	75,8 ± 5,3	<0,01*
A peak (cm/s)	37,7 ± 4,9	46,1 ± 5,9	<0,05*
IVRT (ms)	80,1 ± 8,7	62,6 ± 24,6	<0,05*
DT (ms)	211,6 ± 31,2	209,3 ± 12,8	NS
E/A	1,8 ± 0,4	1,6 ± 0,2	NS

Legend: *BSA* body surface area, *BMI* body mass index, *HR* heart rate, *IVS* interventricular septum, *PW* posterior wall, *LVEDd* left ventricular end-diastolic diameter, *LVESd* left ventricular end-systolic diameter, *LVCMI* left ventricle cardiac mass index, *Aor* diameter of aortic root, *RV* right ventricular diameter, *EF* ejection fraction. *PP* Pulmonary Pressure, *LA area* Left Atrium Area, *LA Volume* Left Atria Volume, *MAPSE* Mitral annular plane systolic excursion, *TAPSE* Tricuspid annulus plane systolic excursion, *E* ^*1*^E wave, *A* ^*1*^ A wave, *IVRT*, Isovolumic relaxation time, *DT* Deceleration Time, *S* s wave, *E/A* E /A ratio

*Indicates Data with Statistical Differences

The assessment of body composition shows that the soccer players have the lowest body fat percentage while the highest body fat percentage was found in the IS. Hydration status was in the upper limits of the range for all subject investigated. Rugby players had the greatest total body water compared to the IS and the soccer players, predominantly represented by intracellular water.

There were no significant differences in the systolic and diastolic blood pressure mean values of the groups of athletes, if compared to IS, while the HR mean value, at rest condition, in soccer and rugby players was significantly lower than the IS (61.1 ± 9.2/min^soccer^ 66.9 ± 10.5/min^rugby^ vs 78.8 ± 1.9/min^inactive^ p <0.05) (Tables 1, and 3).

### Echocardiographic data of soccer players: comparison with the control group

All the echocardiographic values were within the normal range. No substantial differences in the measurement of the parameters, by the two different cardiologists, were observed The 95% limits of agreement were around the mean value ± 2 *s* The groups of soccer players showed significantly higher mean values than the IS : LVEDd and LVEDd were 53.3 ± 3. 7 mm, and 33.0 ± 3.1 mm for soccer players, while 49.4 ± 5.6 and 30.9 mm ± 3.6 mm for IS with P <0.05. The IVS and PW were respectively 10.1 ± 1.0 mm and 9.8 ± 1.5 mm in soccer players and 9.3 ± 0.7 mm and 9.2 ± 1.1 mm in IS with P <0.05. LVM, LVMi values were, on the contrary, above, greater than the normal range and significantly higher in athletes than in IS (187.1 ± 40.6 g = LVM in soccer players, and 153.4 ± 32.1 g in IS, P <0.05, LVMI = 97.5 ± 25.6 g/m2 compared to 78.8 ± 16.6 g/m2, p <0.05; (Table 1).

The diameter of the right ventricle was, otherwise, not significantly different between the two groups (RV = 23.1 ± 3.5 mm for soccer players and 22.3 ± 2.6 for the IS). The diastolic parameters showed a normal pattern in both the groups. However, despite there are some differences, with higher values in soccers players with respect of inactive subjects (Table 1), they maintained normal, as demonstrated by E/A data in both and with a value around the upper and lower limits of the normal range. No significant difference of the other parameters measured and particularly PP resulted to be normal.

After five years of follow-up, the soccer players did not show any significant changes among the LV parameters, that were still within levels considered normal. In fact the value of LVEDd ranged from 53.35 ± 3.72 to 52.98 mm ± 3.03 mm; the value LVESd from 32.73 ± 2.97 to 32.69 mm ± 2.72 mm and the LVMI from 97.77 ± 18.15 to 95.84 ± 17.92 g/m2 g/m2. Considering the observation study of the echographic follow -up has been conducted at the onset of the seasonal training period, no significant variation has been observed on the LVCMi parameter. No significant change was found also in the diameter of the aortic root which ranged from 30.22 ± 3.41 mm to 32.28 ± 2.88 mm (Table 2). Standard diastolic parameters showed a normal pattern and were maintained in the normal range for all the 5 years.

**Table 2:** Echocardiographic five-year follow-up of the soccer players

	I year	II year	III year	IV year	V year
Age (yrs)	23,0 ± 4,74	24,2 ± 4,71	25,5 ± 4,97	26,3 ± 4,96	27,4 ± 4,88
BSA (m^2^)	1,92 ± 0,12	1,94 ± 0,12	1,95 ± 0,11	1,94 ± 0,14	1,95 ± 0,12
Height (cm)	180,32 ± 6,64	180,92 ± 6,09	181,04 ± 6,26	181,07 ± 6,27	181,11 ± 6,31
Weight (kg)	74,84 ± 7,80	76,33 ± 7,02	76,15 ± 9,63	77,54 ± 6,87	77,0 ± 9,83
HR	61,4 ± 11,78	60,6 ± 11,67	60 ± 11,10	60,2 ± 8,74	60,1 ± 8,58
IVS (mm)	10,08 ± 1,6	10,12 ± 0,88	10,05 ± 0,84	10,23 ± 0,90	10,22 ± 0,91
PW (mm)	9,65 ± 0,89	9,84 ± 0,65	9,80 ± 0,87	9,85 ± 0,79	9,85 ± 0,80
LVEDd(mm)	53,35 ± 3,72	53,36 ± 3,18	53,48 ± 4,91	52,96 ± 2,90	52,98 ± 3,03
LVESd(mm)	32,73 ± 2,97	33,07 ± 2,74	32,97 ± 3,08	32,59 ± 2,95	32,69 ± 2,72
LVCMi(g/m2)	97,5 ± 25,6	98,5 ± 30,6	96,5 ± 20,4	96,4 ± 21,3	97,8 ± 23,26
AoR(mm)	30,22 ± 3,41	33,07 ± 2,74	31,93 ± 2,90	32,21 ± 2,83	32,28 ± 2,88
Atrium (mm)	34,02 ± 6,39	34,69 ± 5,96	35,90 ± 2,50	35,79 ± 2,67	35,76 ± 3,38
RV(mm)	23,11 ± 4,64	22,39 ± 2,72	23,19 ± 2,79	23,47 ± 2,82	23,54 ± 2,96
EF%	67,05 ± 5,04	66,14 ± 3,10	65,03 ± 3,67	65,87 ± 4,12	66 ± 3,64
E peck (cm/s)	63,11 ± 5,13	71,00 ± 9,75	77,11 ± 14,77	81,17 ± 12,95	83,11 ± 4,24
A peak (cm/s)	37,02 ± 4,97	37,78 ± 9,74	44,67 ± 8,12	43,12 ± 15,86	37,53 ± 11,31
DT (ms)	211,67 ± 31,24	203,44 ± 38,27	195,67 ± 41,33	178,09 ± 35,90	165,51 ± 7,78
IVRT (ms)	80,11 ± 8,73	77,12 ± 8,77	76,44 ± 9,43	70,33 ± 10,61	66,18 ± 5,65
E/A	1,87 ± 0,43	1,96 ± 0,52	2,17 ± 1,19	2,73 ± 1,66	2,33 ± 0,59

Legend: *BSA* body surface area, *HR* heart rate, *IVS* interventricular septum, *PW* posterior wall, *LVEDd* left ventricular end-diastolic diameter, *LVESd* left ventricular end-systolic diameter, *LVCMI* left ventricular Cardiac mass index, *Aor* diameter of aortic root, *RV* right ventricular diameter;*% EF* ejection fraction. *E peck* E wave, *A* A peck, *DT* Deceleration Time, IVRT, Isovolumic relaxation time, wave, *E/A* E/A ratio

### Echocardiographic data of rugby players: comparison with the control group

In rugby players all the echocardiographic parameters were within the normal range despite being significantly higher than in the IS : LVEDd and LVESd (respectively 50.4 ± 4.4 mm and 32.5 ± 4.2 mm and 49.4 ± 5.6 in rugby players and 30.9 mm ± 3.6 mm for IS, *P* <0.05), IVS and PW were 9.5 ± 1.0 mm and 9.6 ± 0.9 mm in rugby players and 9.3 ± 0.7 mm and 9.2 ± 1.1 mm in IS with *P* <0.05 (Table 3).

**Table 3:** Comparison of the values of the rugby players and the IS

	Rugby (A)	IS (B)	*P* Value
	*n* = 114	*n* = 200	
Age (years)	23,7 ± 5,8	26,9 ± 3,7	NS
Height (cm)	178,1 ± 8,6	174,9 ± 5,8	<0,05*
Weight (kg)	86,9 ± 14,9	80,3 ± 12,2	NS
BMI (kg/m^2^)	27,1 ± 1,9	26,3 ± 3,1	<0,05*
BSA (m^2^)	2,05 ± 0,1	1,96 ± 0,2	<0,05*
SBP (mmHg)	124,4 ± 14,4	127,2 ± 11,5	<0,05*
DBP (mmHg)	76,7 ± 5,6	78,8 ± 1,4	<0,05*
HR (b/min)	66,9 ± 10,5	78,8 ± 1,9	<0,05*
IVS (mm)	9,5 ± 1,0	9,3 ± 0,7	<0,05*
PW (mm)	9,6 ± 0,9	9,2 ± 1,1	<0,05*
LVEDd (mm)	50,4 ± 4,4	49,4 ± 5,6	<0,05*
LVESd (mm)	32,5 ± 4,2	30,9 ± 3,6	<0,05*
LVM (g)	158,1 ± 30,5	153,4 ± 32,1	<0,05*
LVCMi (g/m^2^)	81,5 ± 18,6	78,8 ± 16,6	<0,05*
AoR	30,1 ± 3,4	29,5 ± 2,2	NS
EF%	65,4 ± 8,3	62,5 ± 8,4	NS
Atrium	34,2 ± 8,6	31,5 ± 7,6	NS
RV (mm)	22,2 ± 3,5	22,3 ± 2,6	NS
PP (mmhg)	19,2 ± 2,5	19,2 ± 2,2	NS
LA area (cm^2^)	18,8 ± 2,3	17,3 ± 1,5	NS
LA volume (ml)	54,9 ± 15,6	44,3 ± 0,7	NS
MAPSE (mm)	20,0 ± 4,2	18,2 ± 2,6	NS
TAPSE (mm)	28,3 ± 3,2	25,1 ± 2,5	NS
E^1^ (cm/s)	15,2 ± 3,1	14,0 ± 2,4	<0,05*
A^1^ (cm/s)	9,2 ± 2,3	7,3 ± 1,0	NS
S (cm/s)	11,1 ± 3,4	8,22 ± 1,0	<0,05*
E peck (cm/s)	86,4 ± 11,1	75,8 ± 5,3	<0,05*
A peak (cm/s)	55,0 ± 12,9	46,1 ± 5,9	<0,05*
IVRT (ms)	69,8 ± 10,2	62,6 ± 24,6	<0,05*
DT (ms)	193,1 ± 27,3	209,3 ± 12,8	NS
E/A	1,6 ± 0,2	1,6 ± 0,2	NS

Legend: *BSA* body surface area, *BMI* body mass index, *HR* heart rate, *IVS* interventricular septum, *PW* posterior wall, *LVEDd* left ventricular end-diastolic diameter, *LVESd* left ventricular end-systolic diameter, *LVCMI* left ventricle cardiac mass index, *Aor* diameter of aortic root, *RV* right ventricular diameter, *EF* ejection fraction. *PP* Pulmonary Pressure, *LA area* Left Atrium Area, *LA Volume* Left Atria Volume, *MAPSE* Mitral annular plane systolic excursion, *TAPSE* Tricuspid annulus plane systolic excursion, *E* ^*1*^ E wave, *A* ^*1*^ A wave, IVRT, Isovolumic relaxation time, *DT* Deceleration Time, *S* s wave, *E/A* E /A ratio

*Indicates Data with Statistical Differences

After 5 years of follow-up, the RV diameters were not significantly modified in the two groups (RV = 22.2 ± 3.5 mm and 22.3 ± 2.6 in rugby players and IS). LVM and LVMI values were, on the contrary, significantly greater in rugby players if compared to IS (LVM = 158.1 ± 30.5 g and 153.4 ± 32.1 P <0.05; LVMI = 81.5 ± 18.6 g/m2 compared to 78.8 ± 16.6 g/m2, p <0.05). Diastolic parameters were normal I bot the groups and without any significant differences.

During the five years of follow up the rugby players did not show significant differences in the size of LV, which maintained the normal range. The value of LVEDd ranged from 50.43 ± 4.39 to 52.32 mm ± 3.23 mm ± 4.24 mm, the LVESd from 32.51 to 32.8 ± 4.16 mm; LVMI from 81.5 ± 18.6 g/m2 to 87.34 ± 17.92 g/m2 with p = NS for all; also the diameter of the aortic root which varied between 30.68 ± 6.04 and 30.95 ± 2.15 mm) did not show any significant variance (Table 4).

**Table 4:** Echocardiographic five-year follow-up of the rugby players

	I year	II year	III year	IV year	V year
Age (yrs)	22,7 ± 5,8	23,2 ± 5,74	23,9 ± 5,98	24,5 ± 6,32	25,11 ± 6,39
BSA (m^2^)	2,05 ± 0,1	2,03 ± 0,12	2,04 ± 0,11	2,04 ± 0,14	2,04 ± 0,12
Height (cm)	178,1 ± 8,64	177,5 ± 6,44	178,2 ± 6,36	178,2 ± 7,07	178,11 ± 6,98
Weight (kg)	86,19 ± 14,76	85,41 ± 13,24	85,28 ± 13,3	85,56 ± 13,26	86,22 ± 13,75
HR	66,95 ± 10,48	72,3 ± 7,11	73,5 ± 6,02	72,54 ± 5,29	71,1 ± 6,21
IVS (mm)	9,53 ± 1,03	9,67 ± 1,18	9,98 ± 1,0	9,73 ± 1,0	9,79 ± 0,98
PW (mm)	9,63 ± 0,88	9,71 ± 1,02	9,90 ± 0,96	9,64 ± 0,90	9,73 ± 0,94
LVEDd(mm)	50,43 ± 4,39	50,16 ± 3,46	50,01 ± 3,50	52,04 ± 3,59	52,32 ± 3,23
LVESd(mm)	32,51 ± 4,24	32,24 ± 3,39	32,06 ± 3,9	32,63 ± 3,88	32,8 ± 4,16
AoR(mm)	30,68 ± 6,04	31,05 ± 3,11	31,05 ± 2,04	31,17 ± 2,57	30,95 ± 2,15
LVCMi (g/m^2^)	81,5 ± 18,6	82,4 ± 15,5	83,5 ± 20,6	81,2 ± 17,5	81,9 ± 23,6
Atrium (mm)	33,75 ± 6,63	34,85 ± 5,95	35,04 ± 6,67	34,46 ± 6,36	35,65 ± 5,78
RV(mm)	22,26 ± 3,49	21,39 ± 3,31	22,75 ± 4,11	23,46 ± 3,79	23,0 ± 3,86
EF%	63,11 ± 7,52	66,33 ± 1,10	66,16 ± 4,62	65,67 ± 3,50	67,14 ± 4,09
E peck (cm/s)	86,45 ± 11,15	92,33 ± 10,65	88,89 ± 21,57	93,17 ± 12,12	104,05 ± 15,58
A peak (cm/s)	55,09 ± 12,96	56,11 ± 14,34	55,22 ± 14,15	55,04 ± 16,40	58,25 ± 14,70
DT (ms)	193,18 ± 23,38	172,11 ± 41,62	191,56 ± 15,06	188,33 ± 32,36	183,09 ± 19,90
IVRT (ms)	69,81 ± 10,25	71,67 ± 9,27	73,18 ± 10,17	78,05 ± 12,51	77,92 ± 8,71
E/A	1,62 ± 0,28	1,73 ± 0,35	1,66 ± 0,42	1,79 ± 0,44	1,82 ± 0,45

Legend: *BSA* body surface area, *HR* heart rate, *IVS* interventricular septum, *PW* posterior wall, *LVEDd* left ventricular end-diastolic diameter, *LVESd* left ventricular end-systolic diameter, *LVMI* left ventricular mass index, *AoR* diameter of aortic root, *RV* right ventricular diameter, *EF* ejection fraction, *A* A wave, *E* E wave, *DT* Deceleration Time, *IVRT* Isovolumic relaxation time

### Comparison of anthropometric and echocardiographic parameters between professional soccer players’ and rugby players’ groups

During the 5-year follow-up, the study group of soccer players and rugby players did not show any significant differences in the mean systolic and diastolic blood pressure values.

The HR mean value was significantly lower in soccer players (mean value of 61.1 ± 9.2/min) if compared to rugby players (66.9 ± 10.5/min) (Table 5). Body mass index (BMI), body surface area (BSA) and weight were significantly greater in the rugby players if compared to the soccer players (Table 5). In both groups, the left ventricular values were within the normal range. In the group of soccer players only the average values of LVEDd and IVS were significantly higher than in the rugby players (LVEDd 53.3 ± 3.7 mm vs 50.4 ± 4.4; IVS 10.1 ± 1.0 mm vs 9.5 ± 1.0 mm with *P* <0.05) while LVESd and PW were substantially consistent (LVESd 33.0 ± 3.1 mm vs LVESd 32.5 mm ± 4.2 mm and PW 9.8 ± 1.5 mm vs 9.6 ± 0, 9 mm with p = NS.)

**Table 5:** Comparison of the values of the soccer players and the rugby players

	Soccer (A)	Rugby (B)	*P* Value
	*n* = 250	*n* = 60	
Age	24,7 ± 5,6	23,7 ± 5,8	NS
Height(cm)	180,0 ± 6,3	178,1 ± 8,6	NS
Weight (kg)	75,5 ± 7,7	86,9 ± 14,9	<0,05*
BMI (kg/m^2^)	23,3 ± 1,8	27,1 ± 1,9	<0,05*
BSA (m^2^)	1,95 ± 0,1	2,0 ± 0,1	<0,05*
SBP (mmHg)	119,1 ± 7,4	124,4 ± 14,4	NS
DBP (mmHg)	75,4 ± 6,1	76,7 ± 5,6	NS
HR (b/min)	61,1 ± 9,2	66,9 ± 10,5	<0,05*
IVS (mm)	10,1 ± 1,0	9,5 ± 1,0	<0,05*
PW (mm)	9,8 ± 1,5	9,6 ± 0,9	NS
LVEDd (mm)	53,3 ± 3,7	50,4 ± 4,4	<0,05*
LVESd (mm)	33 ± 3,1	32,5 ± 4,2	NS
LVM (g)	187,1 ± 40,6	158,1 ± 30,5	<0,05*
LVMi (g/m^2^)	97,5 ± 25,6	81,5 ± 18,6	<0,05*
AoR	30,8 ± 1,9	30,1 ± 3,4	NS
EF%	67,2 ± 9,5	65,4 ± 8,3	NS
Atrium	34,8 ± 6,1	34,2 ± 8,6	NS
RV (mm)	23,1 ± 3,5	22,2 ± 3,5	NS
PP (mmhg)	18,2 ± 2,5	19,2 ± 2,5	NS
LA area (cm^2^)	19,3 ± 3,4	18,8 ± 2,3	NS
LA Volume (ml)	59,7 ± 4,6	54,9 ± 15,6	NS
MAPSE (mm)	21,8 ± 1,7	20,0 ± 4,2	NS
TAPSE (mm)	28,8 ± 5,8	28,3 ± 3,2	NS
E1 (m/s)	12,2 ± 2,1	15,2 ± 3,1	<0,05*
A1 (m/s)	8,3 ± 2,7	9,2 ± 2,3	NS
S (m/s)	8,3 ± 1,7	11,1 ± 3,4	<0,05*
E peck (cm/s)	63,1 ± 5,1	86,4 ± 11,1	<0,001*
A peak (cm/s)	37,7 ± 4,9	55,0 ± 12,9	<0,001*
IVRT (ms)	80,1 ± 8,7	69,8 ± 10,2	<0,05*
DT (ms)	211,6 ± 31,2	193,1 ± 27,3	NS
E/A	1,8 ± 0,4	1,6 ± 0,2	NS

Legend: *BSA* body surface area, *BMI* body mass index, *HR* heart rate, *IVS* interventricular septum, *PW* posterior wall, *LVEDd* left ventricular end-diastolic diameter, *LVESd* left ventricular end-systolic diameter, *LVCMI* left ventricle cardiac mass index, *Aor* diameter of aortic root, *RV* right ventricular diameter, *EF* ejection fraction. *PP* Pulmonary Pressure, *LA area* Left Atrium Area, *LA Volume* Left Atria Volume, *MAPSE* Mitral annular plane systolic excursion, *TAPSE* Tricuspid annulus plane systolic excursion, *E* ^*1*^: E wave, *A* ^*1*^ A wave, IVRT, Isovolumic relaxation time, *DT* Deceleration Time, *S* s wave, *E/A* E /A ratio

*Indicates Data with Statistical Differences

The RV diameter was not significantly different between the groups (RV = 23.1 ± 3.5 mm to 22.2 ± 3.5 mm in soccer players and in rugby players respectively).

Left ventricular mass and left ventricular mass index were significantly greater in soccer players than in rugby players (LVM = 187.1 ± 40.6 g to 158.1 ± 30.5 g, *P* <0.05, LVMI = 97.5 ± 25.6 ± 18.6 g/m2 compared to 81.5 g/m2, *p* <0.05). The analysis of the results during follow-up showed that the values of LVM and LVMi remained constant without any significant change in 5 years within each group analyzed (Table 1).

On the contrary, the diameter of the left ventricle (LVEDd and LVESd) was significantly greater in both groups of athletes if compared to the IS and the LVEDd parameter increased particularly in the soccer players The behavior of these parameters during follow-up was similar to previous years (Table 2).

The same trend, with significantly higher values in the groups of athletes, was found by considering the results of wall thickness (IVS and PW) of the left ventricle.

Particularly the diastolic parametrs resulted to different in the groups of athletes with respect of of IS. As expected, considering the high aerobic and static component of soccer and rugbists respectively if compared to IS, the different diastolic pattern can be partially justified.

Some of the standard diastolic values are in fact strongly related to the blood pressure and load charge in consequence of the regular training.

**Fig. 1:**
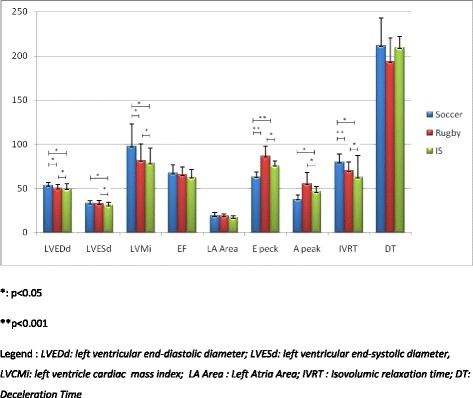
Standard echocardiographic parameters of the 3 groups of subjects investigated (Soccer, rugbyst and inactive subjects)

The principal echo data analyzed in athletes and IS, are summarized in the Fig. 1.

No correlation was found between BMI and LVMI in either group of athletes (*R* = 0.11 between BMI and LVMI of rugby players, *R* = −0.89 in soccer players).

## Discussion

Despite the adaptations of athlete’s heart to training have been already described in several articles in the past, a recent update of the literature dedicated the echo guidelines for the correct approach of the assessment of the measurements of the myocardial chambers, have been produced [12]. The athlete’s heart remain the main topic of many investigations in the imaging modality and the continuing developments in technology have provided new insight in cardiac adaptation in presence of divergent training pattern [13]. Both morphological body surface area patterns associated to a training adaptations, contribute to interpret the athlete’s heart data. The normal systolic and diastolic function is the basis of the characteristics in athlete’s heart to distinguish from the pathological systolic and diastolic impairment [14]. The combination of the standard echocardiography with the new echocardiographyc technologies allow a complete assessment of the all myocardial chambers, like left atrium and right ventricle, normally involved in the heart’s remodeling.

The morphological and functional changes of the heart of trained athletes have been studied by echocardiography since 1975 by Morganroth [1]. Two main types of athlete’s heart, a consequence of regular sport-related training, are described: eccentric myocardial hypertrophy due load, and concentric hypertrophy due to resistance-load. The major characteristic of the former is the increase in dimension of the LV with respect to its thickness, while the main characteristic of the latter is an increase of the prevailing wall thickness not related to an increase in the diameter of the left ventricle [15, 16].

With Magnetic Resonance Spence [3] has recently provided a new description of the morphological aspects of the athletes heart. The LV remodelling pattern observed shows a substantially similar aspect of the cavity size and wall thickness, both in the case of resistance as well training, focusing on and specifying that resistance training may not acutely increase LV systolic wall stress. These observations concern a limited short period of training. Some aspects has been, in addition, reported in literature about the relationship of the morphological aspects of the athlete’s heart with respect of cardiomyopaties [15, 16].

A recent acquisition in a large cohort of athletes followed during their seasonal training [17] has showed that some specific modifications of the main echo parameters, can be found among the elite soccer player followed for a long time. No data are anyway available in literature in the context of other kind of sports as rugby, that cannot be considered similar for its peculiar myocardial impact. In this term, the present investigation can be considered a pilot study. In addition, some important aspects regarding the evidence of an impact the Fatty Free mass parameter in determining the LVCM value, is emerging. This aspect could be a very important future field of interest in Sports Medicine, where some slight but progressive and continous modifications of the body composition, on the basis of the training, kind of sports and nutritional aspects, are influenced and can have at the same time an impact on the heart. In this context also the behaviour of the LVCMi data need to be reinterpreted expecially in the context of sports where the team’s composition is so different like in case of the rugby and where the dynamic component is lower of the static one.

Our results, has been obtained both in basal conditions and from a long-term follow-up with echocardiographic study. The trend of the echocardiographic parameters is in agreement with that reported in the literature [18]. So, with a particular attention to the data reported in a long term follow up [18], it is important to underline that our investigation does not substantially differ for the results reported.

In the first case all values of the morphological parameters of the myocardium remained within the upper normal range. In addition, from the data obtained no substantial morphological differences were found, with the exception of slight differences. This aspect is particularly important considering that it occurred in two different sports with different workload components and therefore in agreement with Spence’s hypothesis.

Despite the fact that with respect to the control group, there was the evidence of a and significant increase in diameter of the left ventricle, associated with a slight increase in wall thickness mainly of the interventricular septum, also with of a significant decrease in heart rate associated to and with evident a specific remodeling of the right ventricle in consequence of a predominant activity of aerobic type in the the elite soccer players, no significant differences were found with respect to the rugby players.

In essence, the two groups had a similar trend regarding cardiac parameters, independently of the anthropometric parameters evaluated by the specific and validated measurement [8–15]. In fact, during the 5-year follow-up, the correlation index R which studies the tendency of a variable to vary as the function of an other, showed no positive correlation between the two parameters. At present, the absence of a significant correlation (R <1) between the values of LVMI and the anthropometric parameters does not absolutely excludes the possibility that some parameters, expressing cardiac morphological changes, especially in the group where BMI was particularly high, i.e. the rugby players, can be specially derived from this aspect. Future study will be necessary to deep investigate this aspect.

A special consideration needs for the correct interpretation of the diastolic function in athletes. As expected there are some significant differences were found in athletes if compared to the IS. This regard the principal standard diastolic echo parameters, but also the TDI parameters. All the data are within the normal range, however the differences found, can highlight the potential impact of the training in this physiological phase on the myocardial revolution where the role of the pressure and blood loading impact is predominant with respect of the morphological mo modifications.

Further studies are necessary for a better and correct interpretation, using for example non-traditional methods such as strain, strain rate, TDI, or speckle tracking methods.

From our results we found aspects in line with the literature. The heart of an athlete is basically normal in size, although its dimensions are within the upper limits of normality. However, thanks to a long-term follow-up new data have emerged confirming that this aspect is maintained over time, at constant workout, despite some differences in the anthropometric parameters.

## Conclusions

The principal aspect of the present study is the confirmation of the uniformity of the morphological parameters in the groups of athletes from sports of mixed workload component, without any particular configuration in eccentric or concentric shape of the athlete’s heart.

Despite the fact that soccer players and rugby players have significantly different BSA, BMI and weight, the results show that the morpho-functional cardiac values depend mainly on the type and intensity of training performed.

There are certain aspects and components characterizing the heart of an athlete that could not be studied in this context, as well as genetic factors and gender differences.

A longer follow-up would be advantageous for defining more accurately physical characteristics of athletes of different sports, and it could show closely the boundary between physiological and pathological modifications, despite these two sports are classified, according to the same criteria, in the different class, such as soccer and rugby in the scale of JACC 2005 [4].

The physiological cardiac adaptations in eccentric or concentric shape as a result of physical training, detected by different authors, could therefore be considered specific in consequence of the different study design.

### Limitations of the study

Although the number of athletes considered in the study, in particular rugby players, is not particularly high, it can be considered statistically significant for the results relating to the morphological characteristics of athletes.

## Abbreviations

AoR: Diameter of aortic root
BMI: Body mass index
BSA: Body surface area
EF: Ejection fraction
HR: Heart rate
IVS: Interventricular septum
LVEDd: Left ventricular end-diastolic diameter
LVESd: Left ventricular end-systolic diameter
LVMI: Left ventricular mass index
PW: Posterior wall
RV: Right ventricle
